# Supplementary material for: The Distribution and Spread of Susceptible and Resistant Neisseria gonorrhoeae Across Demographic Groups in a Major Metropolitan Center
Source: Clin Infect Dis. 2020 Aug 23;73(9):e3146–55. doi: 10.1093/cid/ciaa1229 (PMC8563204; doi:10.1093/cid/ciaa1229)
Supplement: ciaa1229_suppl_Supplementary_Materials [file ciaa1229_suppl_supplementary_materials.docx]

**Title:** The distribution and spread of susceptible and resistant *Neisseria gonorrhoeae* across demographic groups in a major metropolitan center

**Authors:** Tatum D. Mortimer, Preeti Pathela, Addie Crawley, Jennifer L. Rakeman, Ying Lin, Simon R. Harris, Susan Blank, Julia A. Schillinger, Yonatan H. Grad

Supplementary Material

[Supplementary Text 2](#_Toc46404263)

[Supplementary Table 4. Clustering of isolates with 12 SNP cutoff and addition of nested isolates. 2](#_Toc46404264)

[References 3](#_Toc46404265)

[Supplementary Figures 4](#_Toc46404266)

[Supplementary Figure 1. Clustering of *N. gonorrhoeae* isolates from New York City. 4](#_Toc46404267)

[Supplementary Figure 2. Genetic distance between isolates in patients with multiple samples. 5](#_Toc46404268)

[Supplementary Figure 3. The association between MICs and sexual behavior is explained by lineage differences for ceftriaxone and azithromycin. 6](#_Toc46404269)

[Supplementary Figure 4. MSM race/ethnicity is associated with ciprofloxacin MICs. 7](#_Toc46404270)

# Supplementary Text

*Defining transmission clusters.* We did not have information on sex partner links in our patient population, so we could not define a SNP distance cutoff consistent with sexual partnerships. While a 12 SNP cutoff, proposed based on time scaled phylogenies [1], is consistent with the median time between collection dates of our samples (7.7 months), other studies of transmission have used a cutoff of 10 SNPs to identify probable transmission events based on SNP differences between named partners [2]. On clustering isolates based on the more conservative SNP distance (10 SNPs), we observed that isolates nested within a cluster on the phylogeny were not included in the cluster using our method because the genetic distances between the isolate and other members of the cluster were greater than the SNP cutoff (Supplementary Figure 1). One explanation for increased SNP distances could be that the specimen from which the isolate was cultured was collected at a different time compared to other isolates in the cluster, and therefore, presumably a greater number of mutations had accumulated than our SNP cutoff. However, we found that 74% of nested isolates or clusters had specimen collection dates within the range of the cluster. Manual inspection of these isolates showed that the genomes contained unique recombination events identified by Gubbins [3]; the median number of unique recombination blocks for singleton isolates nested within a cluster was 5 (IQR: 0.25-9), compared to a median of 0 (IQR: 0-3) for the entire dataset. We observed SNPs at the boundaries of these events or additional clusters of homoplastic SNPs not identified as recombinant, which was inflating SNP distances from the rest of the cluster (Supplementary Figure 1). Thus, we included any isolate descended from the most recent common ancestor (MRCA) of a cluster for final clustering (Supplementary Figure 2). Clustering with a 12 SNP cutoff did not substantially change the results of clustering analyses (Supplementary Table 4).

# Supplementary Table 4. Clustering of isolates with 12 SNP cutoff and addition of nested isolates.

| Observation from 10 SNP cutoff | Value with 12 SNP cutoff |
| --- | --- |
| Clusters >= 10 samples show evidence of bridging | 8/13 large clusters with evidence of bridging |
| Association between sexual behavior groups and clustering | p-value = 0.0003278 |
| Association between heterosexual race/ethnicity and clustering | p-value = 0.01723 |

# References

1. De Silva D, Peters J, Cole K, et al. Whole-genome sequencing to determine transmission of Neisseria gonorrhoeae: an observational study. The Lancet Infectious Diseases **2016**; 16:1295–1303.

2. Williamson DA, Chow EPF, Gorrie CL, et al. Bridging of Neisseria gonorrhoeae lineages across sexual networks in the HIV pre-exposure prophylaxis era. Nat Commun **2019**; 10:1–10.

3. Croucher NJ, Page AJ, Connor TR, et al. Rapid phylogenetic analysis of large samples of recombinant bacterial whole genome sequences using Gubbins. Nucl Acids Res **2015**; 43:e15–e15.

# Supplementary Figures


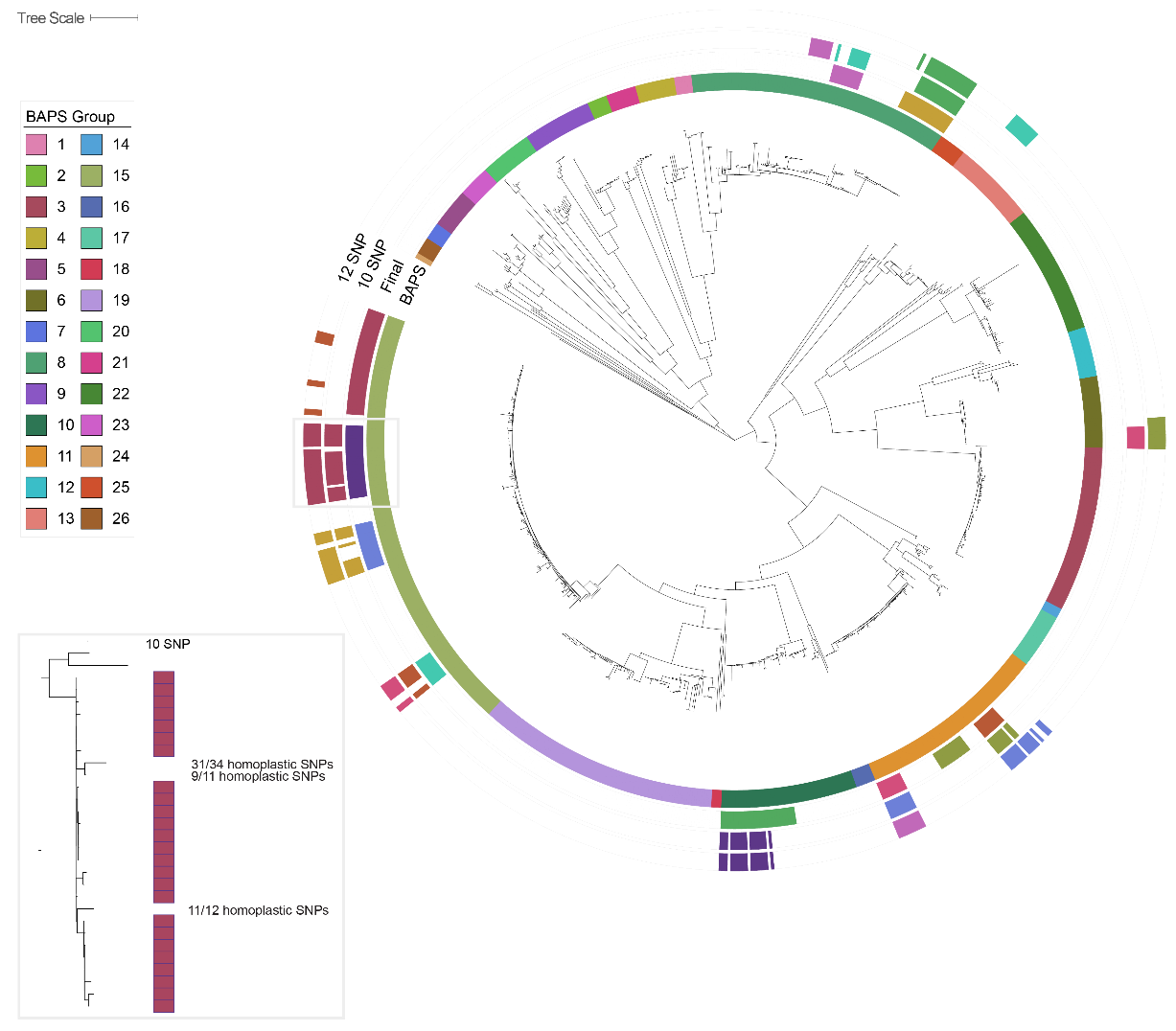


Supplementary Figure 1. Clustering of *N. gonorrhoeae* isolates from New York City. The innermost annotation ring corresponds to clusters identified using a phylogeny-based analysis in fastbaps. The second annotation ring corresponds to the 10 largest final transmission clusters defined using a 10 non-recombinant SNP cutoff with any additional nested isolates. The third and fourth annotation rings correspond to the 10 largest transmission clusters using 10 non-recombinant SNP and 12 non-recombinant SNP cutoffs, respectively. Without correcting for undetected recombination (see inset), transmission clusters were missing nested isolates. **Inset.** A portion of the phylogenetic tree corresponding to final cluster 1 (dark purple in Supplementary Figure 1 annotation ring) is shown on the left. Black boxes correspond to isolates considered part of the cluster using a 10 non-recombinant SNP cutoff. Isolates nested within the phylogeny but with distances greater than 10 SNPs have homoplastic SNPs contributing to their distance, which may represent undetected recombination events.


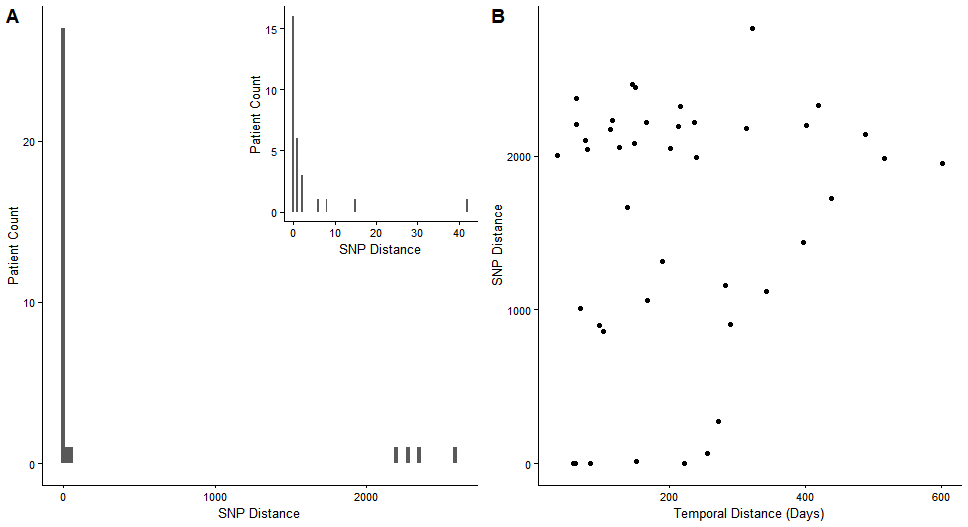


Supplementary Figure 2. Genetic distance between isolates in patients with multiple samples. **A)** Patients with multiple samples collected at the same visit can be infected with multiple strains. The non-recombinant SNP distance between isolates collected on the same day is shown on the X axis, and the number of patients is shown on the Y axis. 18.2% of patients are infected by multiple strains. **B)** Patients who return to the clinic are infected by new strains. The temporal distance between clinic visits is plotted on the X axis, and the non-recombinant SNP distance between isolates is plotted on the Y axis. 90.5% of observed re-infections were with a new strain.


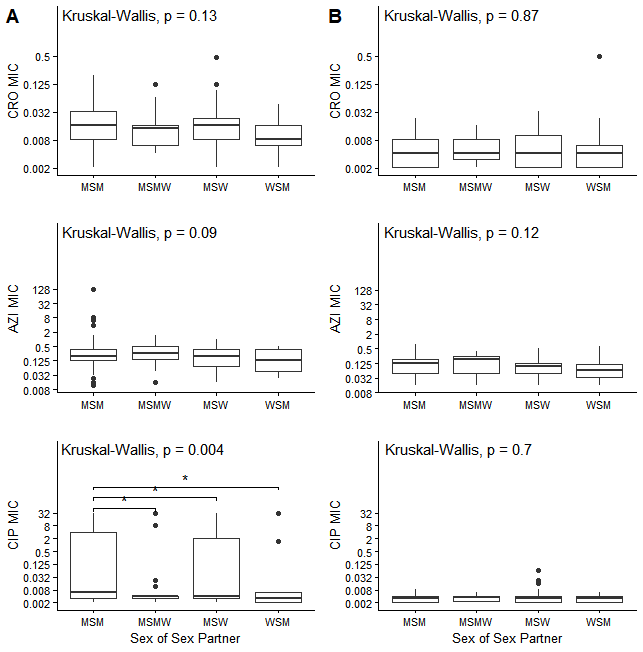


Supplementary Figure 3. The association between MICs and sexual behavior is explained by lineage differences for ceftriaxone and azithromycin. A) Lineage A B) Lineage B Significant pairwise comparisons are denoted by a bracket and asterisks (* p < 0.05).


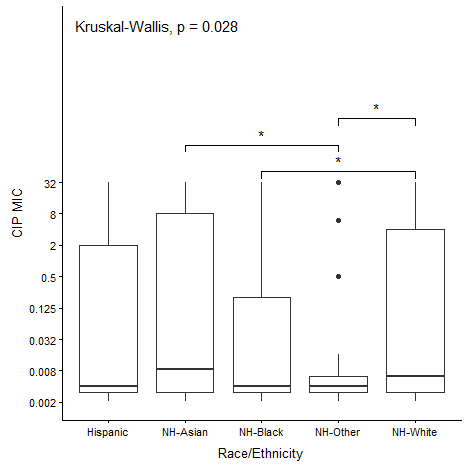


Supplementary Figure 4. MSM race/ethnicity is associated with ciprofloxacin MICs. Significant pairwise comparisons are denoted by a bracket and asterisks (* p < 0.05).
